# The Modulation of Water, Nitrogen, and Phosphorous Supply for Growth Optimization of the Evergreen Shrubs *Ammopiptanthus mongolicus* for Revegetation Purpose

**DOI:** 10.3389/fpls.2021.766523

**Published:** 2021-12-17

**Authors:** Rana Roy, M. Golam Mahboob, Carmen Arena, Md. Abdul Kader, Shirin Sultana, Ahmed Khairul Hasan, Jinxin Wang, Tanwne Sarker, Ruiqi Zhang, Milon Barmon

**Affiliations:** ^1^College of Natural Resources and Environment, Northwest A&F University, Yangling, China; ^2^Department of Agroforestry & Environmental Science, Sylhet Agricultural University, Sylhet, Bangladesh; ^3^ASICT Division, Bangladesh Agricultural Research Institute, Gazipur, Bangladesh; ^4^Department of Biology, University of Naples “Federico II”, Complesso Universitario Monte S. Angelo, Naples, Italy; ^5^School of Agriculture, Geography, Environment, Ocean and Natural Sciences, University of the South Pacific, Suva, Fiji; ^6^Department of Soil Science, Bangladesh Agricultural University, Mymensingh, Bangladesh; ^7^College of Science, Health, Education and Engineering, Murdoch University, Murdoch, WA, Australia; ^8^Open School, Bangladesh Open University, Gazipur, Bangladesh; ^9^Department of Agronomy, Bangladesh Agricultural University, Mymensingh, Bangladesh; ^10^Key Laboratory of Plant Nutrition and the Agri-Environment in Northwest China, Ministry of Agriculture, Yangling, China; ^11^School of Economics and Finance, Xi'an Jiaotong University, Xi'an, China; ^12^Institute of Soil and Water Conservation, Northwest A&F University, Yangling, China; ^13^Key Laboratory of Vegetation Restoration and Management of Degraded Ecosystems, South China Botanical Garden, Chinese Academy of Sciences, Guangzhou, China

**Keywords:** *Ammopiptanthus mongolicus*, coal mine spoils, chemical fertilizers, revegetation, water-shortage

## Abstract

Surface mining is a critical anthropogenic activity that significantly alters the ecosystem. Revegetation practices are largely utilized to compensate for these detrimental impacts of surface mining. In this study, we investigated the effects of five water (W) regimes [W_40_: 40%, W_48_: 48%, W_60_: 60%, W_72_: 72%, and W_80_: 80% of field capacity (FC)], five nitrogen (N) (N_0_: 0, N_24_: 24, N_60_: 60, N_96_: 96, and N_120_: 120 mg kg^−1^ soil), and five phosphorus (P) fertilizer doses (P_0_: 0, P_36_: 36, P_90_: 90, P_144_: 144, and P_180_: 180 mg kg^−1^ soil) on morpho-physiological and biochemical parameters of *Ammopiptanthus mongolicus* plants to assess the capability of this species to be used for restoration purposes. The results showed that under low W-N resources, *A. mongolicus* exhibited poor growth performance (i.e., reduced plant height, stem diameter, and dry biomass) in coal-degraded spoils, indicating that *A. mongolicus* exhibited successful adaptive mechanisms by reducing its biomass production to survive long in environmental stress conditions. Compared with control, moderate to high W and N-P application rates greatly enhanced the net photosynthesis rates, transpiration rates, water-use efficiency, chlorophyll (Chl) *a*, Chl *b*, total Chl, and carotenoid contents. Under low-W content, the N-P fertilization enhanced the contents of proline and soluble sugar, as well as the activities of superoxide dismutase, catalase, and peroxidase in leaf tissues, reducing the oxidative stress. Changes in plant growth and metabolism in W-shortage conditions supplied with N-P fertilization may be an adaptive strategy that is essential for its conservation and restoration in the desert ecosystem. The best growth performance was observed in plants under W supplements corresponding to 70% of FC and N and P doses of 33 and 36 mg kg^−1^ soil, respectively. Our results provide useful information for revegetation and ecological restoration in coal-degraded and arid-degraded lands in the world using endangered species *A. mongolicus*.

## Introduction

Surface mining is the most common technique for coal mining worldwide, although its use has been highly criticized due to its various negative environmental impacts, including changes in topography, land degradation, and loss of biodiversity (Simmons et al., 2008; Józefowska et al., 2017). Besides its detrimental impact on both the vegetation cover and parameters of soil quality, coal mining generates spoil material (commonly known as “coal mine spoil”), turning the mine sites into deserts (Xiao et al., 2020). The northwestern region of China has abundant coal reserves (Fan et al., 2018), contributing around 70% of overall coal output of China. As a result of this excessive exploitation of mining resources, serious ecological and environmental disturbances have developed (Xiao et al., 2020). Therefore, the use of economically viable and effective techniques for the restoration of degraded mining sites would be desirable from a sustainability standpoint.

Ecological restoration practices such as revegetation of mine-degraded areas have gathered significant attention in the last decade (Khamzina et al., 2008; Cui et al., 2019). This technique has been extensively used to prevent desertification and land degradation (Ahirwal et al., 2017; Mukhopadhyay et al., 2017), especially in places where the adverse properties of the resulting mine spoil soils impair or completely prevent the normal process of revegetation through ecological succession (Feng et al., 2019). Nutrients essential for plant growth like nitrogen (N) and phosphorus (P) are typically low in coal mine spoils (Roy et al., 2020a), limiting plant growth and development, as plants grown in these substrates spend a considerable amount of energy to survive and a less amount of energy in expressing their potential productivity (Merwad et al., 2018). Coal mine spoils are also drought-prone and, thus, drought-resistant native and leguminous species are often preferred for revegetation purposes in these soils (Bungart and Hüttl, 2001; Maiti, 2013). Native plant species can colonize the degraded areas over time, thus increasing the genetic diversity and the aesthetic value of regenerated areas (Ahirwal and Maiti, 2017; Ahirwal et al., 2017). Moreover, leguminous species can better survive in nitrogen-deficient soils than non-leguminous species (Yang et al., 2016), and they can also increase soil fertility through the N fixation process (Diatta et al., 2020). *Ammopiptanthus mongolicus* is a broadleaf leguminous shrub naturally distributed in the northwestern China, where scarcity of soil water and very low availability of N and P limit the survival of this and other plant species (Gao et al., 2006; Shen et al., 2015; Yang et al., 2015). Judicious use of soil water, N and P resources, as well as the employment of suitable plant species, are determinants for the success of revegetation projects in coal-mined areas such as northwestern China (Hu et al., 2015).

Previous studies have demonstrated that water and nutrients may have a cumulative and synergistic effect on plant growth, affecting not only their photosynthetic performance but also their agronomical value (Cao et al., 2021). The combined application of water and N-P fertilization is generally used to increase grain yield, vegetable quality, and fruit properties (Liu et al., 2019) and to enhance water-use efficiency (WUE) (Liu et al., 2013). However, less is known about the impact of the combined application of water and N-P fertilization on the growth of leguminous shrub species such as *A. mongolicus* planted on coal mine spoil. Filling this gap of information could be useful to propose *A. mongolicus* as a suitable species for vegetation restoration in water-limited coal mine areas, based on the potential that legumes have survived multiple stresses (Nguyen et al., 2020). Thus, the objective of this study was to assess the effects of various water regimes and N-P fertilization rates on morphophysiological and biochemical traits of *A. mongolicus* plants established on coal mine spoils and to find the optimum water and N-P application rate.

## Materials and Methods

A pot trial (under an artificial shed) with 1-year-old *A. mongolicus* plants was performed at the Northwest Agriculture and Forestry University, Yangling, Shaanxi, China. Coal-mine spoil was collected from Lingwu, China, and sampled (Supplementary Table S1) according to our previous study (Roy et al., 2020b). Then, 14 kg of sample material was taken in plastic pots (upper diameter, bottom diameter, and height were 32, 27, and 30 cm, respectively) and 1-year-old *A. mongolicus* plant was transplanted into each pot in early March 2018. Following transplantation, plants were daily watered for 1 month with an average of 2.6 mm water per day (W day^−1^), to compensate the evapotranspiration losses and avoid any water stress, after which different water (W) regimes based on various field capacity (FC) levels and N-P fertilization rates were applied until the end of October 2018. Five different water levels were used to reflect the soil water content at FC (i.e., 80% FC = highest, 72% FC = high, 60% FC = moderate, 48% FC = low, and 40% FC = very-low dose). We did not consider 100% FC, because in arid and semi-arid regions, W scarcity is the major obstacle to crop production, and it is very difficult and expensive to supply enough W for plant growth (Afshar et al., 2014). Therefore, in this study, we set the highest and lowest W levels as 80% and 40% FC, respectively, according to some previous literature (Sun et al., 2009; Shen and Li, 2011; Zhao et al., 2015). Furthermore, five levels of N and P (mg kg^−1^), such as 120.0 and 180.0, respectively (highest), 96.0 and 144.0, respectively (high), 60.0 and 90.0, respectively (moderate), 24.0 and 36.0, respectively (low), and 0 and 0, respectively (no N and P dose), were also applied. The ranges of W, N, and P in this study were chosen based on the previous literature (Sun et al., 2009; Shen and Li, 2011; He et al., 2013; Zhao et al., 2015; Huang et al., 2016). For this, we used a standard response surface methodology (RSM) called central composite design (CCD). In statistical analysis, CCD is the most commonly used fractional factorial design, useful in RSM, for building a second-order (quadratic) model for the response variable without using a complete three-level factorial experiment. In this design, the center points are augmented with a group of axial points called star points. In this study, using CCD, a total of twenty-one treatments were formed (Bhattacharya, 2021). Each treatment had three repetitions (3 × 21 = 63). The detail of the calculations of these treatments and the experimental design used in this study are provided in Supplementary Appendix A and Supplementary Table S2. The pots were weighed every day to calculate water losses through evapotranspiration, and to maintain the specific FC, the necessary amount of water was added (Roy et al., 2021d). During the experimental period, the average irrigation requirements in 40, 48, 60, 72, and 80% FC treatments were 1.07, 1.21, 1.38, 1.57, and 1.69 mm W d^−1^, respectively. The description of the experimental treatments is presented in Supplementary Table S3. The combination of the lowest W dose (40% FC) and without N-P fertilizers (W_40_N_0_P_0_) was referred to as control. The effects of W, N, and P on integrated growth responses (IGPs) of *A. mongolicus* were investigated according to the approach of Roy et al. (2021a) (details are given in the Supplementary Appendix). All analytical grade chemical reagents were provided by Sigma-Aldrich Trading Co., Ltd., Shanghai, China.

### Assessment of Morphological Growth Traits

Plant height (PH) and stem diameter (SD) were measured 6 months after planting. Plant samples were oven-dried at 70°C until they reached a constant mass weight and then weighed to obtain the dry weight (DW) of the whole plant. The root-shoot biomass ratio (R/S) was calculated by dividing root biomass with shoot biomass.

### Assessments of Physio-Biochemical Constituents and Antioxidant Enzyme Activities

The net photosynthetic rate (Pn), transpiration rate (Tr), and WUE (Pn/Tr) were assessed using the Portable Photosynthesis System CIRAS-3 (Amesbury, MA, USA). The contents of chlorophylls (Chl *a*, Chl *b*, and total Chl) and carotenoids (Cars) were estimated according to our previous study (Roy et al., 2020a). Leaf water potential (LWP) was assessed before 6:00 a.m. on upper fully expanded and light-exposed leaves using a PMS-Model 1000 pressure chamber (PMS Instrument Company, Albany, OR, USA). The relative water content (RWC) was calculated as suggested by Bandeppa et al. (2019).

For measuring the superoxide (O${}_{2}^{•-}$), hydrogen peroxide (H_2_O_2_), and malondialdehyde (MDA) contents, the methods suggested by Velikova et al. (2000), Chu et al. (2014), and Roy et al. (2021c), respectively, were used. The electrolyte leakage (EL) was measured based on the study by Lutts et al. (1996).

For the determination of proline (Pro; μmol g^−1^ FW) and total soluble sugar (SS) contents (mg g^−1^ FW), sulfosalicylic acid and anthrone-H_2_SO_4_ methods, respectively, were used (Joseph, 1955; Bates et al., 1973). Antioxidant enzyme activities of superoxide dismutase (SOD), catalase (CAT), and peroxidase (POD) were estimated according to our previous study (Roy et al., 2021c).

### Data Analysis

The single variable effect and their interaction (W × N × P) on various response variables of *A. mongolicus*, as well as the estimate of coefficients, were examined by the analysis of variance (ANOVA). The numerical data presented in the tables and figures are means ± standard errors (SEs) of three replicates for each treatment. The optimum W-N-P rate was identified by applying the desirability function approach of Derringer by using Design Expert statistical software (version 11.0; Stat-Ease, Inc., Minneapolis, MN, USA). Heatmap and principal component analysis (PCA) were carried out according to Roy et al. (2020b).

## Results

### Morphological Attributes

The PH, SD, and DW were positively influenced by different water (W) regimes. Treatment 9, the one that received the highest water content (i.e., W_80_N_60_P_90_), resulted in the highest PH, SD, and DW of *A. mongolicus*, which were 97, 95, and 82% higher than control (i.e., W_40_N_0_P_0_) (Table 1). All the treatments, including a high-W dose (i.e., 72% FC; treatments 1–4), were among the highest values in all cases, significantly increasing the PH (range: 68–87%), the SD (range: 30–67%), and the DW (range: 53–79%) compared with control. Treatment 11 (W_60_N_120_P_90_), with a highest dose of N and moderate doses of both W and P, also increased PH (+63%), SD (+26%), and DW (+62%) compared with control. Treatments with low (48% FC) and moderate (60% FC) W doses and N and P ranging from very low to high (treatments 5, 6, 7, 12, and 13) increased the DW between 22 and 56% without concomitant increase in neither PH nor SD (Table 1). The treatments W_48_N_96_P_36_ and W_40_N_60_P_90_ significantly increased the R/S ratio by 19.0 and 17%, respectively, compared with control (Table 1).

**Table 1:** Interaction effect of water (W), nitrogen (N), and phosphorus (P) on plant height (PH), stem diameter (SD), dry biomass (DW), and root-shoot (R/S) biomass ratio of *A. mongolicus*.

	**Treatments**	**Coded and actual level of factors**			**PH**	**SD**	**DW**	**R/S**
		**W (% FC)**	**N (mg kg^**−1**^ soil)**	**P (mg kg^**−1**^ soil)**	**cm**	**mm**	**g plant^**−1**^**	
1	W_72_N_96_P_144_	72 (1)	96 (1)	144 (1)	13.21 ± 1.33^abc^	0.72 ± 0.01^b^	5.57 ± 0.12^bc^	0.44 ± 0.01^d^
2	W_72_N_96_P_36_	72 (1)	96 (1)	36 (−1)	13.58 ± 2.86^ab^	0.69 ± 0.01^b^	5.24 ± 0.11^cd^	0.46 ± 0.01^c^
3	W_72_N_24_P_144_	72 (1)	24 (−1)	144 (1)	12.25 ± 1.19^abcde^	0.58 ± 0.01^c^	6.19 ± 0.02^ab^	0.41 ± 0.01^f^
4	W_72_N_24_P_36_	72 (1)	24 (−1)	36 (−1)	12.96 ± 0.73^abcd^	0.56 ± 0.04^cd^	5.4 ± 0.09^c^	0.42 ± 0.01^ef^
5	W_48_N_96_P_144_	48 (−1)	96 (1)	144 (1)	8.78 ± 0.31^fgh^	0.45 ± 0.02^efg^	5.37 ± 0.04^cd^	0.48 ± 0.01^b^
6	W_48_N_96_P_36_	48 (−1)	96 (1)	36 (−1)	9.74 ± 0.38^defgh^	0.41 ± 0.04^g^	4.74 ± 0.13^def^	0.5 ± 0.01^a^
7	W_48_N_24_P_144_	48 (−1)	24 (−1)	144 (1)	7.86 ± 0.57^gh^	0.5 ± 0.03^cdefg^	4.21 ± 0.19^fgh^	0.43 ± 0.01^de^
8	W_48_N_24_P_36_	48 (−1)	24 (−1)	36 (−1)	8.82 ± 0.69^efgh^	0.46 ± 0.02^defg^	3.19 ± 0.11^j^	0.44 ± 0.01^d^
9	W_80_N_60_P_90_	80 (1.68)	60 (0)	90 (0)	14.37 ± 1.78^a^	0.84 ± 0.06^a^	6.27 ± 0.14^a^	0.43 ± 0.01^de^
10	W_40_N_60_P_90_	40 (−1.68)	60 (0)	90 (0)	7.39 ± 0.49^gh^	0.53 ± 0.01^cdef^	3.64 ± 0.02^hij^	0.49 ± 0.01^ab^
11	W_60_N_120_P_90_	60 (0)	120 (1.68)	90 (0)	11.84 ± 1.37^abcdef^	0.54 ± 0.01^cde^	5.6 ± 0.04^bc^	0.48 ± 0.01^b^
12	W_60_N_0_P_90_	60 (0)	0 (−1.68)	90 (0)	10.11 ± 0.34^cdefgh^	0.47 ± 0.01^defg^	5.02 ± 0.09^cde^	0.41 ± 0.01^f^
13	W_60_N_60_P_180_	60 (0)	60 (0)	180 (1.68)	10.09 ± 0.66^cdefgh^	0.5 ± 0.03^cdefg^	5.14 ± 0.04^cd^	0.43 ± 0.01^de^
14	W_60_N_60_P_0_	60 (0)	60 (0)	0 (−1.68)	11.37 ± 1.82^abcdef^	0.45 ± 0.01^efg^	4.07 ± 0.03^ghi^	0.46 ± 0.01^c^
15–20	W_60_N_60_P_90_	60 (0)	60 (0)	90 (0)	10.85 ± 2.53^bcdefg^	0.51 ± 0.05^cdefg^	4.39 ± 0.24^efg^	0.44 ± 0.03^d^
CK	W_40_N_0_P_0_	40 (−1.68)	0 (−1.68)	0 (−1.68)	7.28 ± 0.51^h^	0.43 ± 0.01^fg^	3.45 ± 0.18^ij^	0.42 ± 0.01^ef^

*Different letters in each column are significantly different (p < 0.05) according to LSD test. Each value represents the mean of 3 replications ± SEs*.

### Photosynthetic Parameters and Leaf Water Status

**Figure 1:**
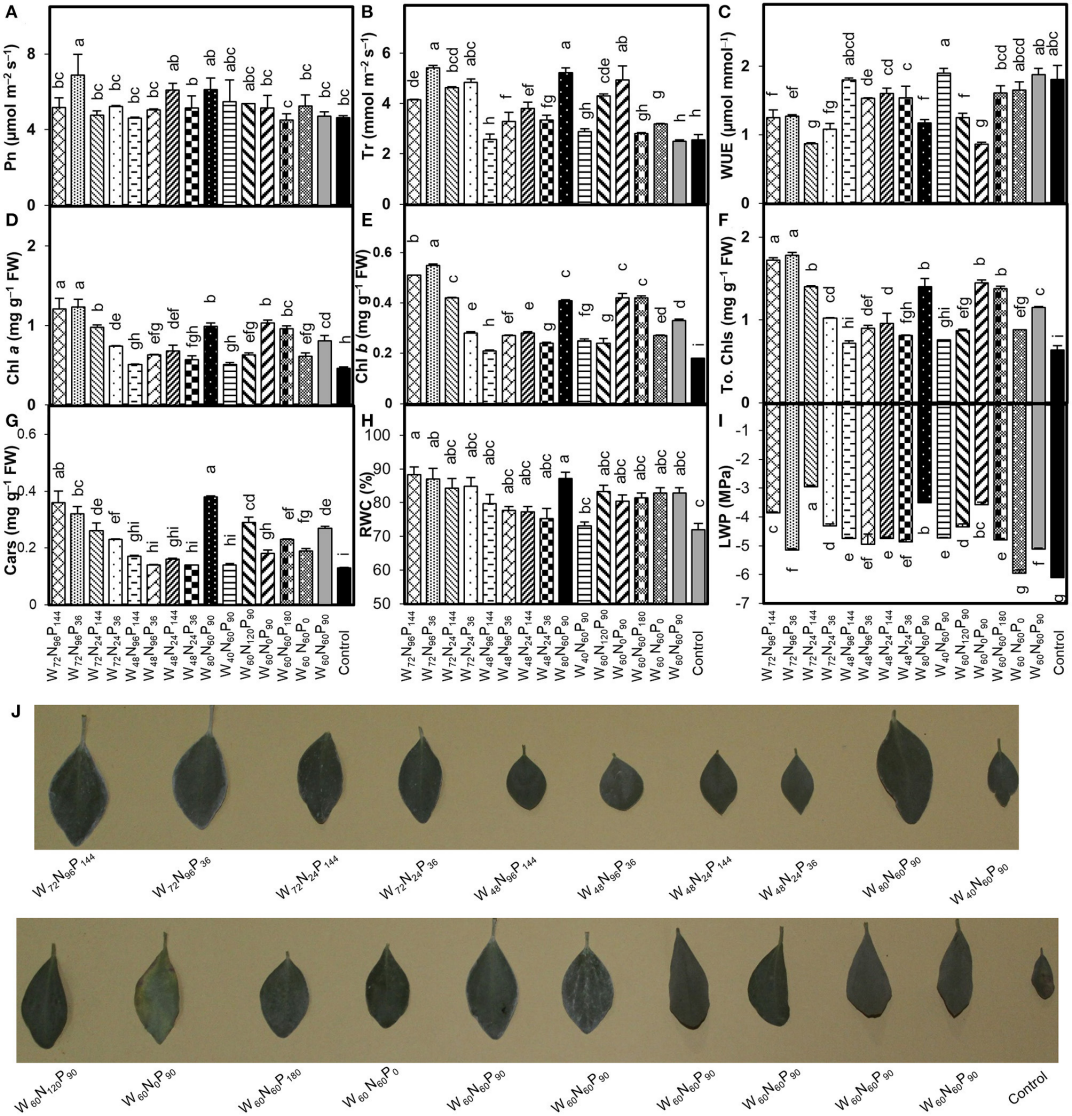
Interaction effect of water (W), nitrogen (N), and phosphorus (P) on the rates of **(A)** net photosynthesis (Pn), **(B)** transpiration (Tr), **(C)** water-use efficiency (WUE), **(D)** chlorophyll (Chl) *a*, **(E)** Chl *b*, **(F)** total Chl, **(G)** carotenoids (Cars) contents, **(H)** relative water content (RWC), **(I)** leaf water potential (LWP) and **(J)** representative individual leaves of *A. mongolicus*. Bars showing the different letters are significantly different (*p* < 0.05) according to LSD test. Each bar represents the mean value (*n* = 3) ± standard error (SE).

Treatments W_72_N_96_P_36_ and W_80_N_60_P_90_ significantly increased (*p* < 0.05) Pn by 48 and 32%, and Tr by 112 and 105%, respectively, compared with control (Figures 1A,B). The WUE of *A. mongolicus* leaves considerably decreased in all treatments compared with control (Figure 1C). The addition of high N and W amounts enhanced the photosynthetic pigment content compared with control, with treatments W_72_N_96_P_144_ and W_72_N_96_P_36_, increasing Chl *a* by 163 and 167%, Chl *b* by 183 and 206%, and total Chl by 169 and 178% over control, respectively (Figures 1D–F).

Treatments W_72_N_96_P_144_ and W_80_N_60_P_90_ increased Cars by 177 and 192%; and RWC by 23 and 21%, respectively, compared with control (Figures 1G,H). LWP under various treatments decreased between 2.6% and 52% compared with control (Figure 1I). Low-W regime ( ≤ 48% FC) induced an evident water-deficiency stress, which together with no or low-N level (0–24 mg kg^−1^ soil) negatively affected the plant growth performance producing chlorosis, necrosis, early senescence, and reduced leaf area (Figure 1J). In contrast, the combination of W (≥60% FC) and N (≥60 mg kg^−1^) remarkably decreased the adverse effects of low-W level, leading to improved performance of *A. mongolicus* (Figure 1J).

### Oxidative Stress Parameters, Osmolytes, and Antioxidant Enzyme Activities

**Figure 2:**
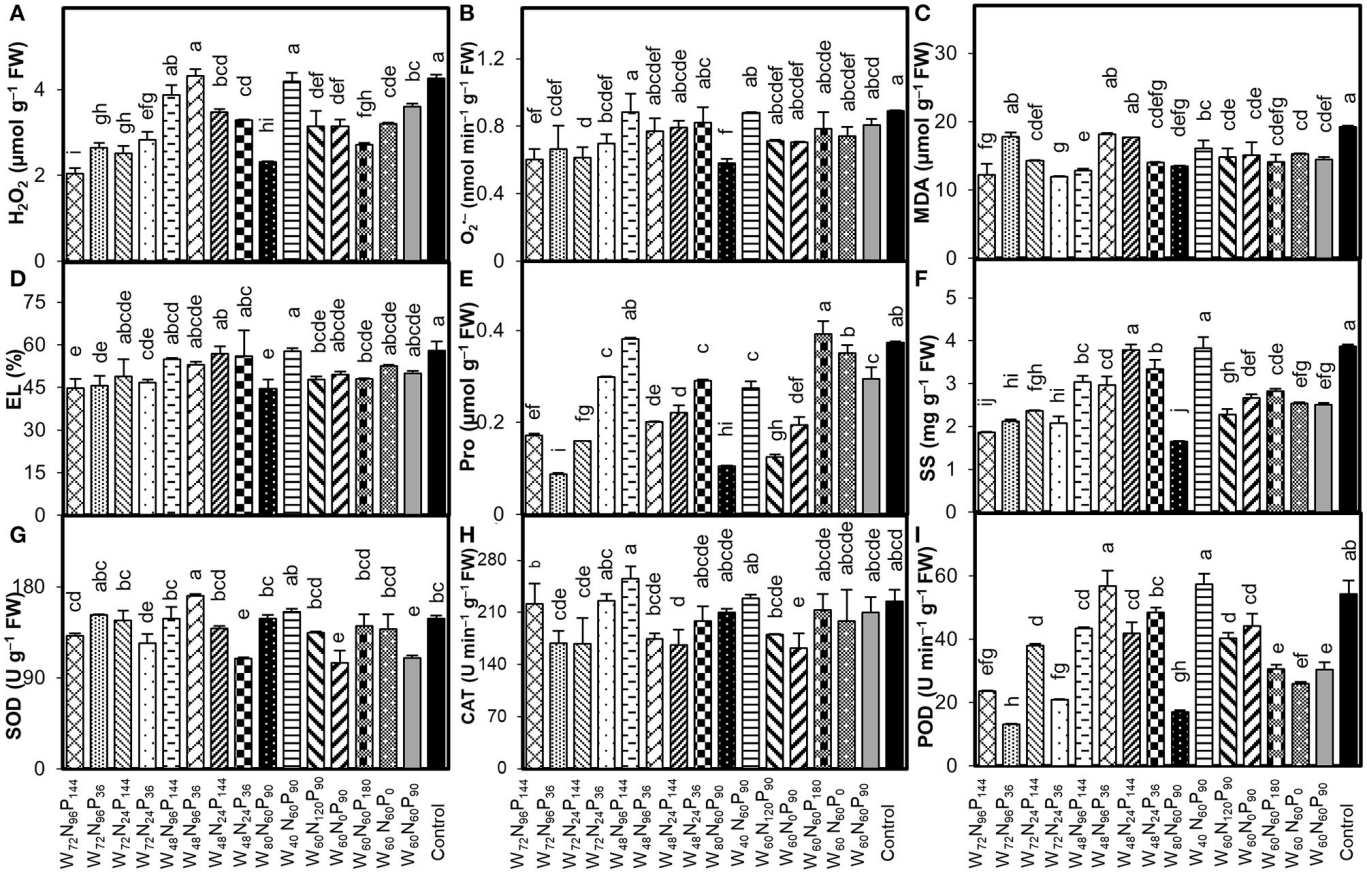
Interaction effect of water (W), nitrogen (N), and phosphorus (P) on **(A)** hydrogen peroxide (H_2_O_2_), **(B)** superoxide anion (O${}_{2}^{•-}$), **(C)** malondialdehyde (MDA), **(D)** electrolyte leakage (EL), **(E)** proline (Pro) content, **(F)** soluble sugar (SS), and activity of **(G)** superoxide dismutase (SOD), **(H)** catalase, (CAT) and **(I)** peroxidase (POD) in *A. mongolicus* leaves. Bars showing the different letters are significantly different (*p* < 0.05) according to LSD test. Each bar represents the mean value (*n* = 3) ± standard error (SE).

Compared with control, treatments W_72_N_96_P_144_ and W_80_N_60_P_90_ reduced the H_2_O_2_ by 52 and 46%, O${}_{2}^{•-}$ by 32 and 35%, and EL by 22.5 and 23.0%, respectively (Figures 2A,B,D). Conversely, the treatment W_40_N_60_P_90_ was not different than control (Figures 2A,B). The addition of W-N-P caused a remarkable decrease in MDA content in all treated plants compared with control. In particular, the treatments W_72_N_24_P_36_ and W_72_N_96_P_144_ decreased the MDA content by 38 and 36%, respectively (Figure 2C). Compared with control, the treatments W_72_N_96_P_36_ and W_80_N_60_P_90_ significantly (*p* < 0.05) reduced the Pro content by 77 and 72% and SS content by 45 and 57%, respectively (Figures 2E,F). The treatment W_60_N_0_P_90_ reduced the SOD and CAT activities by 29 and 28%, respectively (Figures 2G,H). The addition of W ≥60% FC significantly decreased the POD activity, compared with control, regardless of the N-P doses utilized (Figure 2I). Moreover, the treatments W_48_N_96_P_36_ and W_40_N_60_P_90_ did not differ in the CAT activity compared with control.

The positive and negative coefficient values of β_1_, β_2_, and β_3_ (Supplementary Table S3) indicate the synergistic and antagonistic effects of W, N, and P on various growth attributes of *A. mongolicus* (Table 2). The ANOVA analysis showed that synergistic effects resulted from the combined effect of W, N, and P doses on SD, DW, Cars, and RWC but antagonistic effects on EL (Table 2). The ANOVA analysis also showed that W and N application had synergistic effects on PH, Pn, Chl *a*, Chl *b*, and total Chl but antagonistic effects on Pro and SS contents and POD activity. Besides, W and P had synergistic effects on LWP but antagonistic effects on R/S, H_2_O_2_, and MDA contents. Only W had synergistic effects on Tr but antagonistic effects on WUE, O${}_{2}^{•-}$ content, and activities of SOD and CAT (Table 2).

**Table 2:** Parameter coefficients of the regression equation (Y = b_0_+b_1_A+b_2_B+b_3_C+b_12_AB+b_13_AC+b_23_BC+b_11_A^2^+b_22_B^2^+b_33_C^2^) for different growth parameters of *A. mongolicus*.

**Y**	**b_**0**_**	**b_**1**_**	**b_**2**_**	**b_**3**_**	**b_**12**_**	**b_**13**_**	**b_**23**_**	** ${\text{b}}_{1}^{2}$ **	** ${\text{b}}_{2}^{2}$ **	** ${\text{b}}_{3}^{2}$ **
Plant height	10.855	2.090***	0.463***	−0.377***	−0.033^ns^	0.105**	0.043^ns^	0.016^ns^	0.050*	−0.037^ns^
Stem diameter	0.512	0.092***	0.021***	0.016***	0.046***	−0.004^ns^	0.001^ns^	0.059***	−0.006*	−0.014***
Plant dry weight	4.398	0.682***	0.213***	0.335***	−0.436***	−0.066^ns^	−0.106*	0.196***	0.322***	0.073*
Root to shoot ratio	0.446	−0.017***	0.022***	−0.008***	−0.005*	0.002^ns^	−0.001^ns^	0.005**	0.002^ns^	0.001^ns^
Net photosynthesis rate	4.716	0.162***	0.063***	−0.211***	0.455***	−0.335***	−0.323***	0.386***	0.194***	0.065***
Transpiration rate	2.504	0.729***	−0.167***	−0.175***	0.172***	−0.153***	−0.279***	0.555***	0.753***	0.180***
Water use efficiency	1.884	−0.236***	0.104***	0.002^ns^	0.048**	−0.070***	0.050**	−0.125***	−0.295***	−0.092***
Chlorophyll *a*	0.808	0.189***	0.095**	−0.041^ns^	0.041^ns^	0.091**	−0.061^ns^			
Chlorophyll *b*	0.340	0.075***	0.043**	−0.018^ns^	0.018^ns^	0.048**	−0.035*			
Total chlorophylls	1.148	0.264***	0.138**	−0.059^ns^	0.059^ns^	0.139**	−0.096*			
Carotenoids	0.277	0.070***	0.028***	0.014**	0.023***	0.003^ns^	0.002^ns^	−0.007*	−0.017***	−0.026***
Leaf water potential	−5.113	0.372***	−0.227***	0.360***	−0.209***	0.289***	−0.001^ns^	0.351***	0.409***	−0.091***
Leaf relative water content	82.860	4.263***	1.159***	0.166^ns^	0.155^ns^	−0.401^ns^	0.253^ns^	−0.866**	−0.236^ns^	−0.168^ns^
Hydrogen peroxide content	3.602	−0.593***	0.057*	−0.148***	−0.260***	−0.085*	−0.115***	−0.118***	−0.153***	−0.220***
Superoxide anion content	0.807	−0.087***	0.001^ns^	0.001^ns^	−0.010^ns^	−0.029**	0.021*	−0.027***	−0.034***	−0.015*
Malondialdehyde content	14.750	−0.804***	0.198^ns^	−0.501**	0.559**	−0.184^ns^	−2.111***			
Electrolyte leakage	50.335	−4.179***	−0.947*	−0.272^ns^						
Proline content	0.295	−0.048***	−0.018***	0.009*	−0.034***	−0.021***	0.059***	−0.039***	−0.050***	0.025***
Soluble sugar content	2.497	−0.613***	−0.162***	0.073*	0.083*	−0.060^ns^	−0.115***	0.097**	0.001^ns^	0.076*
Superoxide dismutase activity	109.353	−1.771*	9.831***	1.032^ns^	−7.429***	−0.571^ns^	−11.939***	15.341***	3.941***	10.982***
Catalase activity	210.269	−3.175**	6.794***	5.014***	−8.608***	−6.798***	28.093***	2.998**	−14.054***	−1.775^ns^
Peroxidase activity	30.481	−11.912***	−1.391***	1.121***	−3.986***	5.926***	−1.651***	2.282***	4.069***	−0.883***
Integrated growth performances	0.454	0.117***	0.013*	0.016**	0.026***	0.017*	−0.012^ns^	0.001*	0.016**	−0.011*

***p < 0.001, **p < 0.01, *p < 0.05 and ns, not significant; represent significance of effects from ANOVA.

### PCA and Heatmap

**Figure 3:**
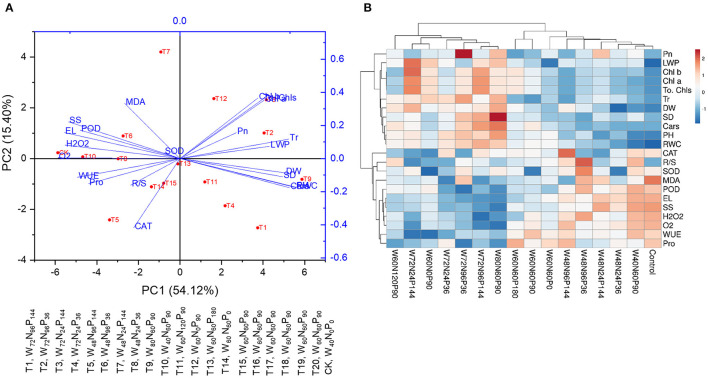
**(A)** Heatmap and **(B)** PCA show the interaction effect of water (W), nitrogen (N), and phosphorus (P) on various growth responses of *A. mongolicus*.

The first five principal components (PCs) of PCA were associated with eigenvalues above one, and the first two PCs explained 69.5% (PC1 = 54.1% and PC2 = 15.4%) of the total variation (Figure 3A). Increases in W doses caused a clear separation of PC1 with the highest W treatment (T9, W_80_N_60_P_90_) positioned on the rightest side and the lowest W treatments (CK, W_40_N_0_P_0_; and T10, W_40_N_60_P_90_) located on the leftist side of the PC1; the allocation on the PC2 axis was associated with the efficient use of N (Figure 3A). Treatments W_80_N_60_P_90_ (T9), W_72_N_96_P_36_ (T2), W_72_N_24_P_144_ (T3), W_72_N_96_P_144_ (T1), and W_72_N_24_P_36_ (T4) were positioned in the positive direction of PC1 and positively connected with growth traits (PH, SD, and DW), photosynthetic traits (Pn and Tr), Chl contents, and leaf water content (LWP and RWC) (Figure 3A). Furthermore, treatments with W-regime ≤ 48% FC, namely W_40_N_0_P_0_ (CK), W_40_N_60_P_90_ (T10), W_48_N_96_P_36_ (T6), and W_48_N_24_P_36_ (T8), were positioned in the negative region of PC1 and were associated with a high accretion of reactive oxygen species (ROS) (like H_2_O_2_ and O${}_{2}^{•-}$), osmolytes (Pro and SS), MDA content, and antioxidant enzyme activities (like SOD, POD, and CAT) (Figure 3A).

Heatmap analysis (Figure 3B) showed that *A. mongolicus* control plants and plants subjected to treatments W_40_N_60_P_90_, W_48_N_24_P_36_, W_48_N_24_P_144_, W_48_N_96_P_36_, W_48_N_96_P_144_, W_60_N_60_P_0_, W_60_N_60_P_90_, and W_60_N_60_P_180_ were grouped on the right of the heatmap, united by high levels of MDA, osmolyte accumulation, ROS production, and antioxidant enzyme activities. The treatments with W additions ≥60% FC, namely W_80_N_60_P_90_, W_72_N_96_P_144_, W_72_N_96_P_36_, W_72_N_24_P_36_, W_72_N_24_P_144_, W_60_N_0_P_90_, and W_60_N_120_P_90_, clustered on the left of the heatmap based on low levels of MDA and ROS production and high levels of growth-related traits like photosynthetic traits, Chl contents, and water status (Figure 3B).

### Interactive Effects of W, N, and P on IGP of *A. mongolicus*

**Figure 4:**
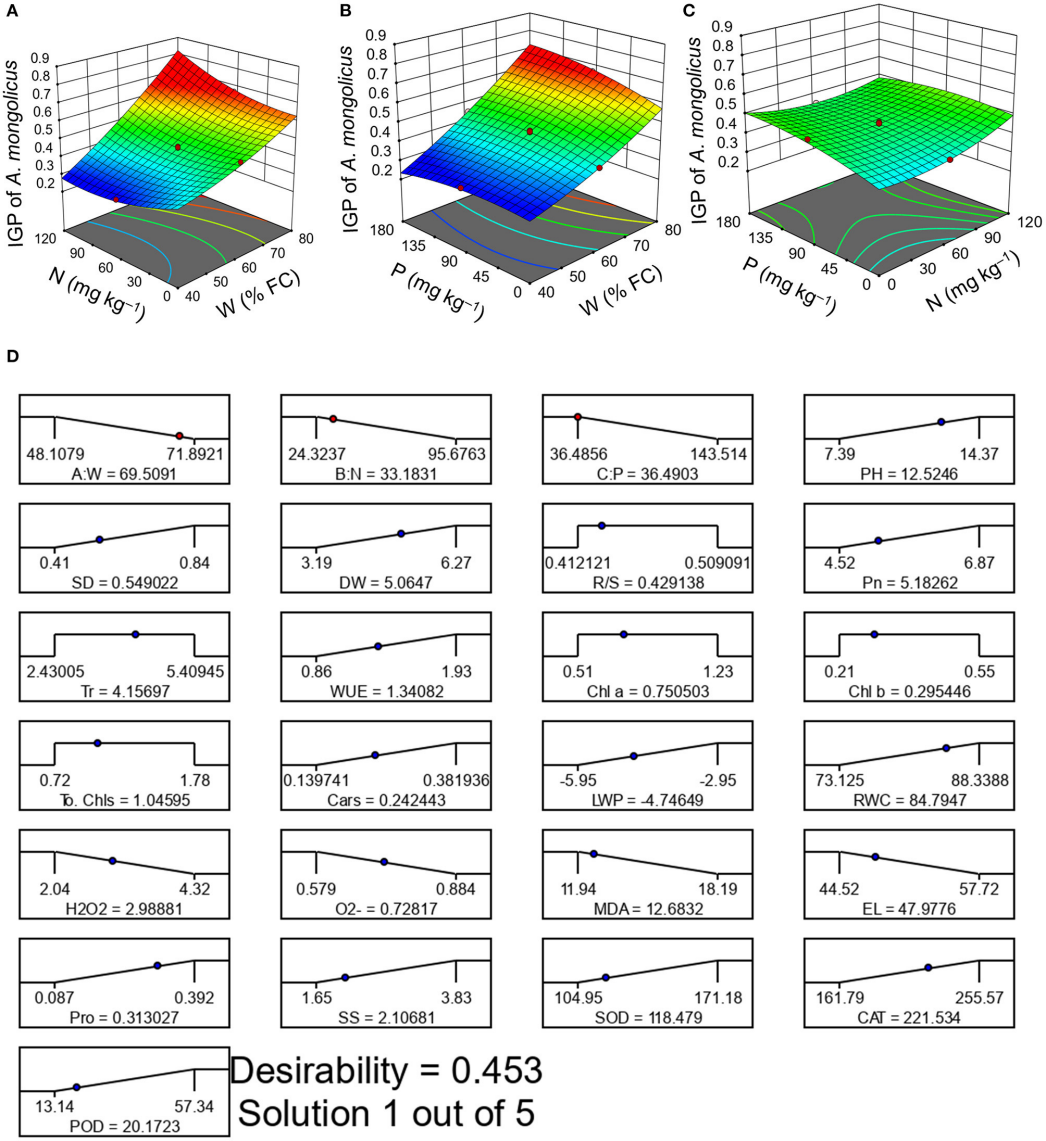
3-D response surface plots presenting the integrated growth performance of *A. mongolicus* **(A)** water (W) × nitrogen (N), **(B)** W × phosphorus (P), **(C)** N × P, and **(D)** desirability ramp presenting the optimal W-N-P rate.

A 3D response surface plot presented interactive effects of W × N (Figure 4A), W × P (Figure 4B), and N × P (Figure 4C) on the IGP of *A. mongolicus*. The treatment W_80_N_60_P_90_ presented the highest IGP of 0.67 (Supplementary Table S4).

Figures 4A,B shows that the IGP of *A. mongolicus* progressively increased with an increase of W application. The IGP of *A. mongolicus* clearly declined at a low-W dose, and the addition of N-P did not have any positive effects on the IGP, indicating that W was the major controlling factor. But, the IGP of *A. mongolicus* evidently advanced with the rise of N-P levels at the highest W supply ( Figures 4A,B). Findings from ANOVA also verify that W-N and W-P interactions have a significant (*p* < 0.01) effect on the IGP of *A. mongolicus* (Table 2). The N × P interaction on the IGP (Figure 4C) suggested that they did not depend on each other and that both had a significant antagonistic effect (*p* < 0.05) on the improvement of growth performance of *A. mongolicus* (Table 2). To identify the optimal W-N-P application rate, we used RSM techniques and found optimum combinations as W at 70% FC, N at 33 mg kg^−1^, and P at 36 mg kg^−1^ (Figure 4D). An excellent correlation between the projected and practical values indicates that the RSM approach is trustworthy (Supplementary Table S5).

## Discussion

The degradation of the ecosystem by coal mining activities is considered a severe environmental problem worldwide. Vegetation establishment in coal mine spoils is possible with the use of appropriate restoration techniques and judicious management of a degraded area. To this end, the selection of the most suitable plant species is one of the most important decisions in restoration practices, as those species should be able to thrive in degraded areas that are poor in plant nutrients and subjected to a combination of environmental stresses. Plants able to counteract environmental constraints and, at the same time, capable of rapid growth with a minimum of resources could represent a suitable investment. Our results show that increments of W doses, irrespective of the N-P fertilizer rates, increased the PH, SD, and DW in *A. mongolicus* plants planted on coal mine spoils. However, growth-associated parameters of *A. mongolicus* dramatically declined when plants were supplied with a W regime ≤ 48% FC. This is possible because under low-W availability, N in the soil did not mobilize well, and plant roots could not uptake N and other nutrients adequately through mass flow mechanism, thus ultimately inhibiting water and nutrient flow through the xylem to the surrounding cells, which reduces the values for different growth attributes (Zhang et al., 2016). Insufficient water availability causes a decrease in cell turgor pressure and limits plant growth (Yang et al., 2020). Maheswari et al. (2007) reported that increasing water stress in plants resulted in several anatomical changes, including a decrease in cell volume and division, intercellular spaces, and cell-wall thickness, which limit the overall plant growth. Remarkably, *A. mongolicus* plants growing under low water and nutrient resource conditions exhibited successful adaptive mechanisms to overcome various stresses and were able to reduce their biomass production in order to survive for longer periods of time (Gao et al., 2006). The synergistic effect of W, N, and P on plant morphological growth was also demonstrated by the ANOVA (Table 2), heatmap, and PCA analyses ( Figures 3A,B).

Among plant physiological processes, photosynthesis is the most vital process important for plant growth (Gururani et al., 2013). The application of N and P fertilizers plays a significant role in improving plant photosynthetic performance, and they are also critical for the synthesis of cytosine and total Chl contents (Roy et al., 2021d). Higher photosynthesis performance is intimately connected to higher plant growth and biomass production, as also confirmed by our study. However, the level of W in the soil may modify the N-P fertilizer uptake by plant roots, control stomatal opening, photosynthetic performance, and biomass accumulation of *A. mongolicus* plants. The reason behind this fact is that an increase of ROS in plant cells under W-shortage conditions increases the degradation of Chl, reducing photosynthetic capacity and growth performance (Ashraf and Foolad, 2007). Jin et al. (2018) reported that reducing growth attributes of *A. mongolicus* shrubs under extreme W deficit and low levels of nutrients is an adaptive mechanism to survive for long periods of time in harsh environments by minimizing stomatal conductance and photosynthesis performance. However, increased WUE at low-W content suggests that *A. mongolicus* has evolved conservative water use strategies such as increasing W-uptake by enlarging root length and decreasing W-losses by reducing Tr to adapt and survive under extreme water deficit conditions (Xu et al., 2002; Musembi et al., 2015).

Results of our study show that W regime ≥60% FC reduced ROS (H_2_O_2_ and O${}_{2}^{•-}$) and EL production in *A. mongolicus* plants, regardless of the N-P rates added; conversely, water FC <60% leads to oxidative stress in the plant through the proliferation of ROS. These results may be due to the excess production of ROS in plants that initiated EL following oxidation of the cell membrane and photosynthetic inhibition due to the alteration in the nature of associated proteins (Venkatesh et al., 2012). The MDA can be used as a proxy to determine the oxidative damage on the cell membrane (Göbel and Feussner, 2009). The decrease in the RWC content and the increase in MDA content under low W and N supply likely suppressed or hindered the capacity of ROS-scavenging antioxidant enzymes, loss of Chl, remobilization of nutrients, and occurrence of membrane damage, with the subsequent reduction in plant growth (Abid et al., 2016; Roy et al., 2020b). This may be the reason for reported decreases in biomass production of *A. mongolicus*, as observed in this study. Therefore, strict control of the production of ROS and MDA contents in the plant cell is necessary for the growth of the plant.

The accumulation of osmolytes such as Pro and SS with decreasing W-availability is associated with an osmotic adjustment of plants under W-stress to maintain the integrity of the cellular membrane under these conditions (Guo et al., 2010; Blum, 2017). Our results show that the appliance of N-P fertilizers under low soil-W supply increased Pro and SS contents and decreased ROS and MDA contents, suggesting that N-P input significantly increased the osmoprotectant levels while decreasing the oxidative damage under W-stress conditions. Shubhra et al. (2004) documented an increase in SS concentration in response to N-P administration under drought conditions, which may have been caused by the reduction of normal SS transport, utilization, and distribution under the low-W regime. In addition, increased Pro synthesis under W-stress and N-P fertilization may be a response to stress; this amino acid acts as a scavenger, supplying osmolytes and energy to stressed tissues and assisting the plant in tolerating stress. Jin et al. (2018) also documented that *A. mongolicus* enhances the Pro content in shoots to survive under severe drought conditions and to reduce ROS levels. It is also reported by several studies that Pro and SS contents increase during W-stress, resulting in an increase in the tolerance of plant to drought stress (Behr et al., 2017; Mohammadi et al., 2018; Roy et al., 2021b,c), as also observed in this study. In general, adaptation to drought stress is dependent on the amount of ROS neutralized by the antioxidant system, which must be kept at a low level (Mascher et al., 2005). Our results demonstrated that the application of N-P fertilizers under a low-W regime considerably reduced the ROS. This may be related to the enhancement of antioxidant enzyme activities (SOD, CAT, and POD) under N-P supply (Figures 2G–I), since N-P nutrition could improve the synthesis and physiological activities of antioxidant enzymes (Khan et al., 2021; Roy et al., 2021a,c). Therefore, the addition of N-P fertilizers under water-limited environments can improve the plant water status, the osmolytes content, and the activity of antioxidant enzymes, favoring the ROS homeostasis (low production of H_2_O_2_ and O${}_{2}^{•-}$) and the protection of cell membrane (lower levels of MDA and EL contents). Similar findings were also observed in other plant species such as *Tamarix chinensis, Amorpha fruticosa*, and *Elaeagnus angustifolia* (Roy et al., 2020a,b, 2021d). Results from the heatmap and PCA analysis further confirmed that H_2_O_2_, O${}_{2}^{•-}$, EL, and MDA contents in the leaves of *A. mongolicus* were negatively correlated with antioxidant enzyme activities treated with N-P fertilizers at low-W content.

Although the highest W and moderate N-P doses (W_80_N_60_P_90_ treatment) resulted in the best growth performance in *A. mongolicus* planted on coal mine spoils, the RSM also represents a satisfactory means of identifying the optimal W-N-P levels for plant growth. Excellent correlation between the projected and practical values among all the responses indicates that the function method provided by RSM may be successfully used to optimize both W and N-P application rates and plant growth. The RSM approach was successfully exercised in several experiments to achieve the finest growth performance for numerous plant species by screening the optimal W-N-P combination (Sepaskhah et al., 2006; Koocheki et al., 2014; Roy et al., 2021a, 2022). Similar to the approach used in this study, which identifies that the most promising combination of levels is W_70_N_33_P_36_, our previous study also utilized the RSM method to find the best W-N-P proportions for maximizing the growth of the species *Elaeagnus angustifolia* planted on coal mine spoils (Roy et al., 2020b).

Under optimum W-N-P doses, *A. mongolicus* exhibits its capability to effectively establish itself in coal mine-degraded spoils by evolving multiple morphological, physiological, and biochemical adaptations, which is the first step of ecological restoration in a degraded area. Moreover, initial revegetation with *A. mongolicus* and appropriate W-N-P doses could boost the fertility of the spoils and could create a favorable condition for the survival of herbs and microorganisms, promoting natural succession (Chu et al., 2012). In agreement with our findings, other researchers have also proposed the use of *A. mongolicus* for revegetation and ecological restoration in arid and semi-arid regions (Zhao et al., 2007; Kleinhesselink et al., 2014; Chu et al., 2015).

## Conclusion

This study showed that morphological, physiological, and biological attributes of *A. mongolicus* were influenced by the application of various regimes of W-N-P doses. The addition of N-P resources enhanced plant growth performance more under high-W regime than under low-W regime. Under the low-W regimes, *A. mongolicus* adopted multiple eco-physiological strategies and biochemical adjustments to counteract the limited water supply. The detrimental effect of W-shortage was reduced by the application of N-P fertilizers, indicated by higher osmolytes (Pro and SS) contents and antioxidant enzyme activities (SOD, CAT, and POD) in leaf tissues, resulting in an improvement of the overall plant physiological status. The enzymatic antioxidant defenses prevent oxidative stress and decrease ROS-induced injuries, as proved by low levels of H_2_O_2_, O${}_{2}^{•-}$, EL, and MDA contents. The RSM approach used in this study provided useful information regarding the suitability of *A. mongolicus* in restoration studies and could be successfully used to identify the best W and N-P combination for maximum growth of *A. mongolicus* plants on coal mine spoils. Our experimental results demonstrated that *A. mongolicus* plants, opportunely supplied with appropriate combinations of W-N-P dose, could be an effective strategy to favor the growth of plant and support ecological restoration in coal mine spoils located in arid and semi-arid areas worldwide.

## Data Availability Statement

The original contributions presented in the study are included in the article/Supplementary Material, further inquiries can be directed to the corresponding author/s.

## Author Contributions

RR: conceptualization, data curation, formal analysis, investigation, methodology, software, visualization, writing—original draft, and writing—review and editing. MM, SS, and MB: software and illustrated graphs and figures. CA and MK: writing—review and editing. AH: software. JW: conceptualization, supervision, validation, writing—review and editing, resources, project administration, and funding acquisition. TS and RZ: investigation and visualization. All authors contributed to the article and approved the submitted version.

## Funding

This work was supported by Research and Innovation Team of Key Technologies for Ecological Protection and Restoration, Quality Improvement and Efficiency Enhancement of the Yellow River Basin of Shaanxi Academy of Forestry Sciences (SXLK2020-0305), National Key Research and Development Program of China (2017YFC0504402), and National Natural Science Foundation of China (31670713).

## Conflict of Interest

The authors declare that the research was conducted in the absence of any commercial or financial relationships that could be construed as a potential conflict of interest.

## Publisher's Note

All claims expressed in this article are solely those of the authors and do not necessarily represent those of their affiliated organizations, or those of the publisher, the editors and the reviewers. Any product that may be evaluated in this article, or claim that may be made by its manufacturer, is not guaranteed or endorsed by the publisher.
