# Supplementary material for: The Modulation of Water, Nitrogen, and Phosphorous Supply for Growth Optimization of the Evergreen Shrubs Ammopiptanthus mongolicus for Revegetation Purpose
Source: Front Plant Sci. 2021 Dec 17;12:766523. doi: 10.3389/fpls.2021.766523 (PMC8719576; doi:10.3389/fpls.2021.766523)
Supplement: Supplementary file 1 [file Data_Sheet_1.docx]

Supplementary Material

**Table S1.** Chemical properties of spoil samples before application of fertilizer.

| **Properties** | **Spoil sample** |
| --- | --- |
| Moisture (%) ^a^ | 2.22 ± 0.11 |
| Water holding capacity (%) ^b^ | 11.176 ± 0.47 |
| Bulk density (g cm^‒3^) ^b^ | 1.48 ± 0.01 |
| Coarse-fine sand (>0.1 mm, %) ^c^ | 93.11 ± 0.48 |
| Very fine sand (0.05-0.1 mm, %) ^c^ | 3.94 ± 0.02 |
| Silt + clay (<0.05 mm, %) ^c^ | 2.95 ± 0.0 |
| pH ^d^ | 7.83 ± 0.07 |
| EC (µS cm^‒1^) ^d^ | 162.3 ± 12.22 |
| Total soluble salt (g kg^‒1^) ^e^ | 0.595 ± 0.03 |
| Soil organic matter (g kg^‒1^) ^f^ | 2.165 ± 0.15 |
| Total nitrogen (g kg^‒1^) ^h^ | 0.157 ± 0.01 |
| Total phosphorus (g kg^‒1^) ^i^ | 0.21 ± 0.01 |
| Available nitrogen (mg kg^‒1^) ^j^ | 6.14 ± 0.59 |
| Available phosphorus (mg kg^‒1^) ^k^ | 1.98 ± 0.09 |
| Available potassium (mg kg^‒1^) ^l^ | 66.88 ± 3.89 |

† Values are means ± standard deviation (*n* = 5). ^a^ Oven dry method; ^b^ Cutting ring technique; ^c^ Water-based elutriation; ^d^ Soil/water ratio 1:2.5, pH meter or microprocessor conductivity meter; ^e^ Conductivity method; ^f^ K_2_Cr_2_O_7_-H_2_SO_4_ method; ^h^ Kjeldahl method, ^i^ HClO_4_-H_2_SO_4_ method; ^j^ Alkaline hydrolysis diffusion technique; ^k^ Olsen (0.5 M NaHCO_3_) extracts, analyzed colorimeter; ^l^ 1 MNH4OAc (pH 7) extracts, analyzed AAS.

The independent variables of this study were coded according to the equation (1) and presented in Table S2:

$X_{i}=\frac{Z_{i-}Z_{i0}}{{\Delta Z}_{i}}$ (1)

Here, $X_{i}$ denotes the coded value of the independent or process variables; $Z_{i}$ indicates the real value of the process variable; $Z_{i0}$ denotes the real value of $Z_{i}$ at the center point; and ${\Delta Z}_{i}$ indicates the step change value.

**Table S2.** Factor levels used in central composite design (CCD).

| Independent variables | Codes | Symbols | Coded and actual values | | | | |
| --- | --- | --- | --- | --- | --- | --- | --- |
|  |  |  | - 1.682 | -1 | 0 | +1 | +1.682 |
|  |  |  | Very low/ no dose | Low dose | Moderate dose | High dose | Highest dose |
| Water (W) (% FC) | A | W | 40 | 48 | 60 | 72 | 80 |
| Nitrogen (N) rate (mg kg^‒1^) | B | N | 0 | 24 | 60 | 96 | 120 |
| Phosphorus (P) rate (mg kg^‒1^) | C | P | 0 | 36 | 90 | 144 | 180 |

The CCD consists of a 2^3^ factorial runs with eight factorial points, six axial points and six replicates at the center points comprising a set of 21 (including a control) experiments as evaluated from Eq. (2) below,

N= 2^n^ + 2n+ n_c_ = 2^3^ + (2 × 3) + 6 = 20 + 1 = 21 (2)

where N represents the total number of experiments, ‘n’ is the number of factors and ‘n_c_’ is the number of replicates at the center points. Each experimental treatment was replicated three times, and a total of 63 plants were randomly arranged in the experimental shed with one plant per pot. Lowest W level (W_40_) without N-P fertilizers (W_40_N_0_P_0_) was treated as control. The detailed description of the experimental treatments, their associated codes and actual W, N, P application levels are presented in Table S3.

**Table S3.** Experimental design and layout of treatment combinations according to the central composite design.

|  | Treatments | Coded level of factors (Eq. 1) | | | Quantity applied | | |
| --- | --- | --- | --- | --- | --- | --- | --- |
|  |  | Soil water (W) | Nitrogen (N) | Phosphorus (P) | W  (% FC) | N  (mg kg^‒1^) | P  (mg kg^‒1^) |
| 1 | W_72_N_96_P_144_ | 1 | 1 | 1 | 72 | 96 | 144 |
| 2 | W_72_N_96_P_36_ | 1 | 1 | -1 | 72 | 96 | 36 |
| 3 | W_72_N_24_P_144_ | 1 | -1 | 1 | 72 | 24 | 144 |
| 4 | W_72_N_24_P_36_ | 1 | -1 | -1 | 72 | 24 | 36 |
| 5 | W_48_N_96_P_144_ | -1 | 1 | 1 | 48 | 96 | 144 |
| 6 | W_48_N_96_P_36_ | -1 | 1 | -1 | 48 | 96 | 36 |
| 7 | W_48_N_24_P_144_ | -1 | -1 | 1 | 48 | 24 | 144 |
| 8 | W_48_N_24_P_36_ | -1 | -1 | -1 | 48 | 24 | 36 |
| 9 | W_80_N_60_P_90_ | 1.682 | 0 | 0 | 80 | 60 | 90 |
| 10 | W_40_N_60_P_90_ | -1.682 | 0 | 0 | 40 | 60 | 90 |
| 11 | W_60_N_120_P_90_ | 0 | 1.682 | 0 | 60 | 120 | 90 |
| 12 | W_60_N_0_P_90_ | 0 | -1.682 | 0 | 60 | 0 | 90 |
| 13 | W_60_N_60_P_180_ | 0 | 0 | 1.682 | 60 | 60 | 180 |
| 14 | W_60_N_60_P_0_ | 0 | 0 | -1.682 | 60 | 60 | 0 |
| 15 | W_60_N_60_P_90_ | 0 | 0 | 0 | 60 | 60 | 90 |
| 16 | W_60_N_60_P_90_ | 0 | 0 | 0 | 60 | 60 | 90 |
| 17 | W_60_N_60_P_90_ | 0 | 0 | 0 | 60 | 60 | 90 |
| 18 | W_60_N_60_P_90_ | 0 | 0 | 0 | 60 | 60 | 90 |
| 19 | W_60_N_60_P_90_ | 0 | 0 | 0 | 60 | 60 | 90 |
| 20 | W_60_N_60_P_90_ | 0 | 0 | 0 | 60 | 60 | 90 |
| 21 | W_40_N_0_P_0_ | -1.682 | -1.682 | -1.682 | 40 | 0 | 0 |

* Treatment nomenclature indicates the amount of W application (e.g., W72 = 72% field capacity), the rate of N application (e.g., N96 = 96 mg kg‒1) and the rate of P application designated as a subscript (e.g., P144 = 144 mg kg‒1).

Effects of different treatments on different growth responses of *A. mongolicus* were analyzed with the second-order polynomial model:

Y = b_0_+b_1_A+b_2_B+b_3_C+b_12_AB+b_13_AC+b_23_BC+b_11_A^2^+b_22_B^2^+b_33_C^2^ (3)

Here, Y represents the response variable; b_0_, b_1_, b_2_ and b_3_ are the interactive regression coefﬁcients; A, B and C are the W, N and P doses, respectively.

Interactive effects of W × N, W × P and N × P on integrated growth performance (IGP) of *A. mongolicus* were obtained by transforming the data related to morphological, physiological and biochemical responses in the following equations (4 and 5).

$$X=\frac{x-x_{min}}{x_{max}-x_{min}} (4)$$

$$X=1-\frac{x-x_{min}}{x_{max}-x_{min}} (5)$$

where $X$ and *x* represent the coded and the average calculated value of each treatment, respectively; *x*_min_ and *x*_max_ represent the minimum and maximum value, respectively, obtained from each parameter and different treatments. To calculate IGP of *A. mongolicus*, we used the equation 4 for those growth indices that were positively correlated with W, N and P application. Equation 5 was used for those growth indices that were negatively correlated with W, N and P application.

**Table S4.** Transformed value of morphological, physiological and biochemicals responses of *A. mongolicus*. Plant height (PH), stem diameter (SD), plant dry weight (DW), root-shoot (R/S) ratio, net photosynthesis rate (Pn), transpiration rate (Tr), water use efficiency (WUE), chlorophyll a (Chl *a*) and b (Chl *b*), total chlorophyll (total Chls), carotenoids (Cars), leaf water potential (LWP), leaf relative water content (RWC), hydrogen peroxide (H_2_O_2_), superoxide anion (O_2_^•‒^), malondialdehyde (MDA), electrolyte leakage (EL), proline (Pro), soluble sugars (SS), superoxide dismutase (SOD), catalase (CAT), peroxidase (POD) and ascorbate peroxidase (APX).

| Trt. | PH | SD | DW | R/S | Pn | Tr | WUE | Chl *a* | Chl *b* | To. Chls | Cars | LWP | RWC | H_2_O_2_ | O_2_ | MDA | EL | Pro | SS | SOD | CAT | POD | IGP |
| --- | --- | --- | --- | --- | --- | --- | --- | --- | --- | --- | --- | --- | --- | --- | --- | --- | --- | --- | --- | --- | --- | --- | --- |
| 1 | 0.54 | 0.58 | 0.68 | 0.38 | 0.28 | 0.54 | 0.31 | 0.8 | 0.86 | 0.9 | 0.8 | 0.74 | 0.52 | 0.92 | 0.78 | 0.76 | 0.81 | 0.75 | 0.92 | 0.47 | 0.36 | 0.8 | 0.659 |
| 2 | 0.57 | 0.53 | 0.6 | 0.5 | 0.64 | 0.89 | 0.32 | 0.57 | 0.63 | 0.65 | 0.65 | 0.42 | 0.49 | 0.7 | 0.69 | 0.18 | 0.78 | 0.99 | 0.82 | 0.24 | 0.68 | 1 | 0.615 |
| 3 | 0.46 | 0.36 | 0.82 | 0.02 | 0.2 | 0.68 | 0.04 | 0.82 | 0.97 | 0.95 | 0.44 | 0.97 | 0.42 | 0.75 | 0.77 | 0.55 | 0.68 | 0.79 | 0.73 | 0.3 | 0.69 | 0.54 | 0.589 |
| 4 | 0.52 | 0.33 | 0.64 | 0.14 | 0.29 | 0.74 | 0.19 | 0.33 | 0.26 | 0.34 | 0.33 | 0.63 | 0.44 | 0.64 | 0.63 | 0.79 | 0.74 | 0.4 | 0.84 | 0.55 | 0.33 | 0.85 | 0.498 |
| 5 | 0.19 | 0.15 | 0.63 | 0.74 | 0.17 | 0.11 | 0.7 | 0.1 | 0.07 | 0.11 | 0.14 | 0.52 | 0.32 | 0.26 | 0.34 | 0.7 | 0.48 | 0.17 | 0.48 | 0.28 | 0.15 | 0.43 | 0.329 |
| 6 | 0.27 | 0.09 | 0.49 | 0.99 | 0.25 | 0.31 | 0.51 | 0.22 | 0.23 | 0.25 | 0.03 | 0.47 | 0.27 | 0.11 | 0.52 | 0.14 | 0.55 | 0.67 | 0.51 | 0.03 | 0.65 | 0.18 | 0.352 |
| 7 | 0.12 | 0.23 | 0.37 | 0.2 | 0.47 | 0.45 | 0.56 | 0.27 | 0.26 | 0.3 | 0.11 | 0.52 | 0.26 | 0.41 | 0.48 | 0.19 | 0.42 | 0.61 | 0.21 | 0.39 | 0.7 | 0.46 | 0.363 |
| 8 | 0.19 | 0.17 | 0.13 | 0.38 | 0.28 | 0.32 | 0.52 | 0.16 | 0.15 | 0.18 | 0.03 | 0.49 | 0.21 | 0.47 | 0.44 | 0.57 | 0.45 | 0.42 | 0.37 | 0.72 | 0.5 | 0.34 | 0.340 |
| 9 | 0.63 | 0.77 | 0.84 | 0.2 | 0.48 | 0.85 | 0.25 | 0.58 | 0.6 | 0.65 | 0.86 | 0.83 | 0.49 | 0.82 | 0.82 | 0.63 | 0.82 | 0.94 | 0.99 | 0.28 | 0.43 | 0.93 | 0.668 |
| 10 | 0.08 | 0.28 | 0.24 | 0.8 | 0.35 | 0.19 | 0.77 | 0.1 | 0.18 | 0.14 | 0.03 | 0.52 | 0.16 | 0.15 | 0.35 | 0.35 | 0.4 | 0.47 | 0.19 | 0.2 | 0.31 | 0.17 | 0.292 |
| 11 | 0.43 | 0.29 | 0.68 | 0.74 | 0.32 | 0.59 | 0.31 | 0.22 | 0.15 | 0.23 | 0.54 | 0.62 | 0.4 | 0.52 | 0.61 | 0.49 | 0.71 | 0.88 | 0.76 | 0.43 | 0.61 | 0.49 | 0.501 |
| 12 | 0.29 | 0.18 | 0.55 | 0.08 | 0.27 | 0.77 | 0.03 | 0.62 | 0.63 | 0.69 | 0.17 | 0.81 | 0.33 | 0.52 | 0.62 | 0.46 | 0.66 | 0.69 | 0.62 | 0.76 | 0.72 | 0.42 | 0.495 |
| 13 | 0.29 | 0.24 | 0.58 | 0.26 | 0.15 | 0.17 | 0.57 | 0.55 | 0.63 | 0.63 | 0.32 | 0.51 | 0.36 | 0.68 | 0.5 | 0.56 | 0.71 | 0.14 | 0.56 | 0.36 | 0.41 | 0.67 | 0.448 |
| 14 | 0.39 | 0.15 | 0.34 | 0.5 | 0.3 | 0.28 | 0.6 | 0.2 | 0.23 | 0.23 | 0.2 | 0.22 | 0.39 | 0.5 | 0.56 | 0.44 | 0.56 | 0.25 | 0.67 | 0.4 | 0.5 | 0.76 | 0.394 |
| 15-20 | 0.35 | 0.25 | 0.41 | 0.35 | 0.19 | 0.09 | 0.76 | 0.4 | 0.41 | 0.45 | 0.5 | 0.43 | 0.39 | 0.36 | 0.46 | 0.52 | 0.64 | 0.41 | 0.68 | 0.71 | 0.42 | 0.68 | 0.448 |
| CK | 0.07 | 0.12 | 0.19 | 0.1 | 0.17 | 0.1 | 0.72 | 0.05 | 0 | 0.04 | 0.02 | 0.18 | 0.13 | 0.13 | 0.33 | 0.03 | 0.39 | 0.19 | 0.17 | 0.28 | 0.34 | 0.23 | 0.181 |

**Table S5.** Statistical parameters from the analysis of variance for the regression models of different growth parameters of *A. mongolicus.*

| Growth parameters | Source | F-value | Sequential p-value | R^2^ | Lack of Fit p-value | Adjusted R² | C.V. % | Adequate Precision |
| --- | --- | --- | --- | --- | --- | --- | --- | --- |
| Plant height | Quadratic | 1195.4 | < 0.0001 | 0.999 | 0.107 | 0.998 | 0.713 | 128.19 |
| Stem diameter | Quadratic | 385.2 | < 0.0001 | 0.997 | 0.242 | 0.995 | 1.41 | 78.36 |
| Plant dry weight | Quadratic | 98.88 | < 0.0001 | 0.989 | 0.038 | 0.979 | 2.42 | 37.91 |
| Root to shoot ratio | Quadratic | 47.38 | < 0.0001 | 0.977 | 0.841 | 0.957 | 1.17 | 25.22 |
| Net photosynthesis rate | Quadratic | 347.7 | < 0.0001 | 0.997 | 0.443 | 0.994 | 0.91 | 71.09 |
| Transpiration rate | Quadratic | 295.31 | < 0.0001 | 0.996 | 0.139 | 0.993 | 2.51 | 45.34 |
| Water use efficiency | Quadratic | 207.52 | < 0.0001 | 0.995 | 0.378 | 0.989 | 2.35 | 41.67 |
| Chlorophyll a | 2FI | 17.11 | < 0.0001 | 0.889 | 0.070 | 0.836 | 9.54 | 15.19 |
| Chlorophyll b | 2FI | 13.36 | < 0.0001 | 0.861 | 0.060 | 0.786 | 9.20 | 14.31 |
| Total Chlorophylls | 2FI | 16.37 | < 0.0001 | 0.883 | 0.059 | 0.829 | 8.08 | 15.17 |
| Carotenoids | Quadratic | 84.49 | < 0.0001 | 0.987 | 0.660 | 0.975 | 4.69 | 29.33 |
| Leaf water potential | Quadratic | 245.62 | < 0.0001 | 0.996 | 0.967 | 0.991 | 1.41 | 64.26 |
| Leaf relative water content | Quadratic | 40.37 | < 0.0001 | 0.973 | 0.144 | 0.949 | 1.07 | 23.09 |
| Hydrogen peroxide content | Quadratic | 86.41 | < 0.0001 | 0.987 | 0.203 | 0.976 | 2.88 | 34.51 |
| Superoxide anion content | Quadratic | 30.47 | < 0.0001 | 0.965 | 0.323 | 0.933 | 3.01 | 18.44 |
| Malondialdehyde content | 2FI | 37.18 | < 0.0001 | 0.945 | 0.627 | 0.919 | 3.25 | 20.97 |
| electrolyte leakage | Linear | 41.51 | < 0.0001 | 0.886 | 0.213 | 0.865 | 2.82 | 22.11 |
| Proline content | Quadratic | 110.80 | < 0.0001 | 0.990 | 0.623 | 0.981 | 4.84 | 34.99 |
| Soluble sugar content | Quadratic | 72.69 | < 0.0001 | 0.985 | 0.422 | 0.971 | 3.65 | 30.56 |
| Superoxide dismutase activity | Quadratic | 157.42 | < 0.0001 | 0.993 | 0.539 | 0.986 | 1.79 | 39.46 |
| Catalase activity | Quadratic | 106.21 | < 0.0001 | 0.989 | 0.267 | 0.980 | 1.72 | 39.44 |
| Peroxidase activity | Quadratic | 1079.18 | < 0.0001 | 0.999 | 0.348 | 0.998 | 1.55 | 116.53 |
| Integrated growth performances | Quadratic | 94.68 | < 0.0001 | 0.988 | 0.292 | 0.978 | 3.40 | 35.56 |
