# Supplementary figures and images for: The Modulation of Water, Nitrogen, and Phosphorous Supply for Growth Optimization of the Evergreen Shrubs Ammopiptanthus mongolicus for Revegetation Purpose
Source: Front Plant Sci. 2021 Dec 17;12:766523. doi: 10.3389/fpls.2021.766523 (PMC8719576; doi:10.3389/fpls.2021.766523)

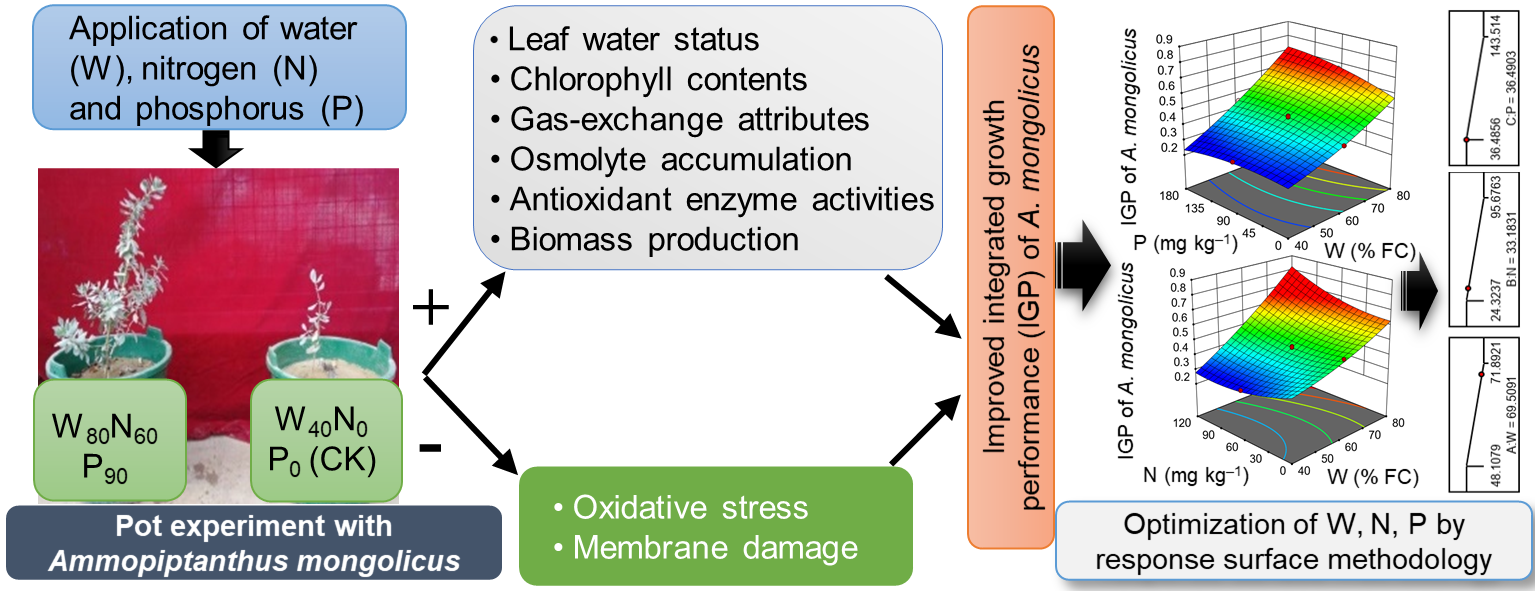

Supplement: Supplementary file 2 [file Image_1.TIF]
